# Case report: Combined cervical incision with an intercostal uniportal video-assisted thoracoscopic surgery approach for mediastinal brachial plexus schwannoma

**DOI:** 10.3389/fonc.2023.1168963

**Published:** 2023-06-12

**Authors:** Linlin Wang, Lihui Ge, Yi Ren

**Affiliations:** ^1^ Department of Thoracic Surgery, Shenyang Chest Hospital and Tenth People’s Hospital, Shenyang, Liaoning, China; ^2^ Department of Health Management, Shengjing Hospital of China Medical University, Shenyang, Liaoning, China

**Keywords:** schwannomas, brachial plexus, uniportal video-assisted thoracoscopic surgery, minimally invasive, faster rehabilitation.

## Abstract

Mediastinal neurogenic tumors primarily originate from the intercostal and sympathetic nerves, whereas schwannomas originating from the brachial plexus are rare. Surgical intervention for such tumors is complex and associated with the risk of postoperative upper limb dysfunction due to their unique anatomical location. In this report, we present the case of a 21-year-old female diagnosed with a mediastinal schwannoma, who underwent a novel surgical approach combining cervical incision and intercostal uniportal video-assisted thoracoscopic surgery (VATS). Our study reviewed the patient’s clinical presentation, treatment approach, pathology, and prognosis. The findings of this study demonstrate that the cervical approach, combined with intercostal uniportal VATS, is a feasible surgical method for the removal of mediastinal schwannomas originating from the brachial plexus.

## 1 Introduction

Schwannomas are benign tumors that originate from the peripheral nervous system. Mediastinal neurogenic tumors account for approximately 20–30% of all mediastinal tumors in adults (1, 2). Mediastinal schwannomas originating from the brachial plexus are extremely rare. These tumors present a challenge for thoracic surgeons because they are located at the top of the thoracic cavity and are closely related to the vertebral body, subclavian artery, and brachial plexus, and carry a risk of postoperative upper limb dysfunction (3). With the improvement of surgical instruments and thoracoscopic techniques, minimally invasive thoracoscopic surgery is the preferred treatment for mediastinal tumors, often combined with a subclavian incision or thoracoscopic “nucleus removal” to treat mediastinal schwannomas (4).

Here, we report a novel method that combines a cervical approach with intercostal uniportal video-assisted thoracoscopic surgery (VATS) exclusively for resection of mediastinal schwannomas of brachial plexus origin.

## 2 Case description

A 21-year-old female presented to the hospital with a mass in the superior mediastinum that was incidentally discovered during a routine physical examination. The patient did not report any symptoms such as chest or shoulder pain, numbness, weakness, or loss of function in the upper limbs, nor did she exhibit any other discomforting clinical manifestations. Further inquiry revealed no history of diabetes, hypertension, or any relevant tumorous diseases. Blood examinations, including tumor markers, biochemistry, complete blood count, arterial blood gas and other pertinent indicators, were unremarkable. Additionally, pulmonary function tests and electrocardiography demonstrated normal results.

**Figure 1:**
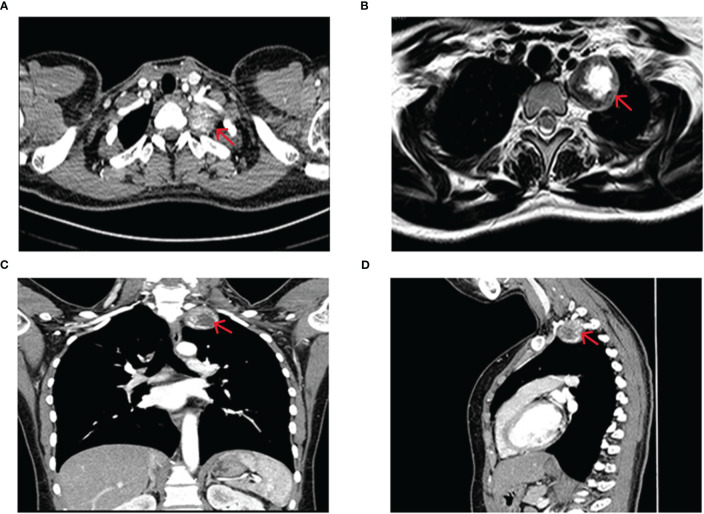
Imaging before surgery. **(A)** CT scan of the chest showing a 33 mm mass with clear boundaries located beside the mediastinum of the upper lobe of the left lung. Calcified nodules within the mass are slightly enhanced. Coronal **(C)** and sagittal **(D)** chest CT images reveal no abnormal changes in the adjacent thoracic vertebrae and ribs. **(B)** MRI of the thoracic vertebrae showing an equal-length T1, and long T2 mainly mixed signal shadows on the left side of the upper mediastinum (around the level of the T1-T2 vertebrae), low-signal shadows are observed on T2WI.

**Figure 2:**
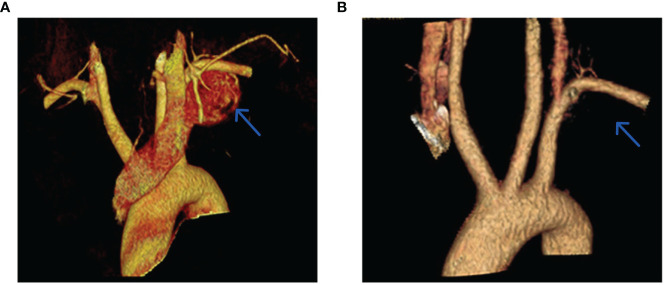
Three-dimensional reconstruction CT scans of tumor vessels were obtained preoperatively **(A)** and postoperatively **(B)**.

Contrast-enhanced computed tomography (CT) of the patient’s chest revealed a well-defined mass, measuring approximately 33mm in diameter, adjacent to the mediastinum of the upper lobe of the left lung (**Figures 1A, C, D**). Three-dimensional reconstruction CT of the tumor blood vessels displayed a distinct boundary between the tumor and the left subclavian artery, with vascular branches visible at the posterior margin of the tumor (**Figure 2A**). Contrast-enhanced magnetic resonance imaging (MRI) of the thoracic vertebrae revealed an equal-length T1 and long T2 signal lesion predominantly located on the left side of the upper mediastinum (approximately at the level of T1-T2 vertebrae), low-signal shadows were detected on T2WI (**Figure 1B**). Based on these imaging characteristics, the diagnosis of schwannoma was highly suspected. The patient declined to undergo positron emission tomography/computed tomography and fine-needle aspiration cytology.

**Figure 3:**
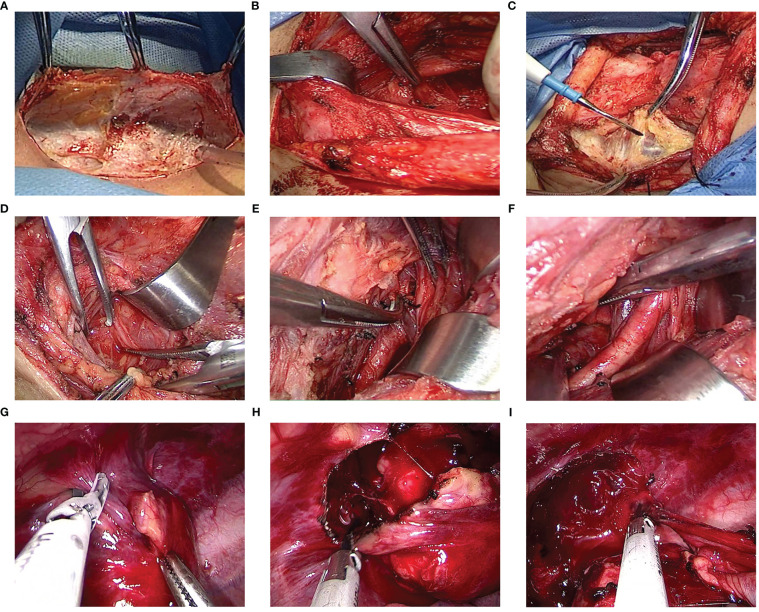
**(A)** The cervical approach incision. **(B–F)** The procedure begins with a horizontal incision along the left supraclavicular area, followed by the sequential removal of tissue layers including the platysma muscle, deep cervical fascia, and carotid sheath, exposing the left recurrent laryngeal nerve, venous horn, lymphatic vessels, and left subclavian artery. The tumor was then carefully separated from the surrounding tissue and the upper pole of the tumor was excised. **(G–I)** The ultrasonic scalpel was utilized to carefully remove the mass from the root of the brachial plexus.

**Figure 4:**
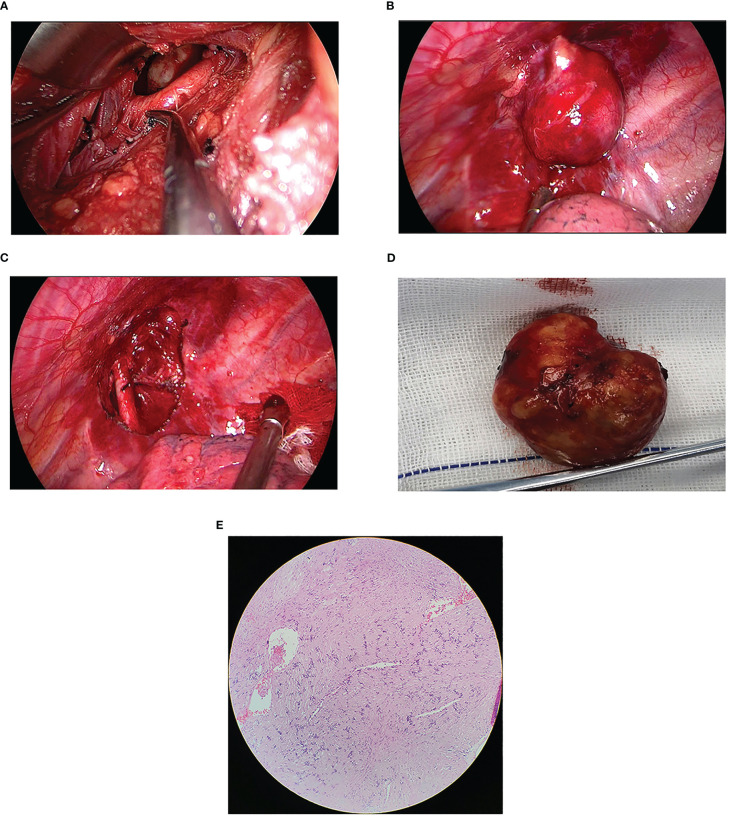
**(A)** The tumor’s anatomical relationship with the subclavian artery and surrounding tissues was visualized from the neck perspective. **(B)** The location of the lesion under uniportal VATS. **(C)** The superior aspect of the thoracic cavity following successful lesion resection. **(D)** The tumor measuring 3.3 × 3.3 cm in diameter after being removed. **(E)** Post-pathology results reveal that the mass was a mediastinal brachial plexus schwannoma. Immunohistochemical analysis revealed positive staining for CD34 (vessel), Ki-67 (approximately 1%), S-100, Vimentin, and SOX-10, whereas staining for CK-P, Desmin, and NSE was negative.

The intricate location of the tumor at the cervicothoracic junction presented a significant challenge of peripheral blood vessels and nerves. To overcome this challenge, a preoperative cervical approach was combined with a uniportal VATS approach to effectively remove the tumor. The surgical equipment used included an ultrasonic scalpel (Ethicon Endo-Surgery, LLC, USA), a 10-mm 30° thoracoscope, operational draw hooks, long curved endoscopic instruments with curved suction and double articulations. The patient was placed under general anesthesia, and a double-lumen endotracheal tube was used for single-lung ventilation. The procedure began with the upper pole of the tumor, and an approximately 8 cm curved incision was made horizontally along the left supraclavicular, extending from the midfront of the neck to the anterior edge of the left sternocleidomastoid muscle (**Figure 3A**). Meticulous dissociation of the platysma muscle, deep cervical fascia, and carotid sheath was performed, exposing the left recurrent laryngeal nerve, venous horn, lymphatic vessels, and left subclavian artery (**Figures 3B–E**). The tumor was then carefully separated from the surrounding tissue and the upper pole of the tumor was excised (**Figure 3F**), revealing its origin from the left brachial plexus. Bleeding was carefully managed, and a thin drainage tube was placed in the neck (**Figure 4A**). Next, the lower pole of the tumor was addressed through a single intercostal incision, with the patient placed in the complete lateral position. A 3.5 cm incision was made along the anterior axillary line in the 4th costal space. The mass was located in the uppermost mediastinum, adjacent to the spine (**Figure 4B**). The mass was carefully removed from the root of the brachial plexus using an ultrasonic scalpel to minimize the risk of peripheral nerve and vascular injury (**Figures 3G–I**). Ultimately, successful removal of the tumor was achieved (**Figures 4C, D**), and the wound was meticulously closed layer by layer. A chest tube was inserted into the posterior part of the incision at the end of the operation, and ropivacaine was administered to the wound for infiltration. Following the procedure, the patient was transferred to the recovery room for monitoring, pain management, and wound care. On the first postoperative day, a chest X-ray was conducted to evaluate lung expansion and the presence of gas or fluid in the pleural cavity.

The surgical procedure lasted approximately 150 minutes, with a blood loss of 30 mL. The cervical drain continued for three days, while the chest drain was carried out for four days. The patient was hospitalized for a total of seven days. The postoperative pathology results revealed that the mass located in the left upper mediastinum was a schwannoma (**Figure 4E**). Postoperatively, the patient experienced a lower skin temperature on the left side than the right side, and diminished sensation in the left upper limb, while normal sensation was observed in the right upper limb. However, the patient’s sensation in the left limb returned to normal after one month. During telephone follow-up, the patient did not report any chest pain for three months after discharge. Three-dimensional reconstruction CT revealed the disappearance of the mass (**Figure 2B**).

## 3 Discussion

Schwannomas are common posterior mediastinal tumors that often originate from the sympathetic or intercostal nerves, but rarely from the brachial plexus (5). Preoperative diagnosis is difficult, and most cases require surgery to determine the nerve source. Due to the complex anatomical position around the tumor and interlacing of nerves and blood vessels, especially the tumor invading the vertebral body, thoracotomy is often required. Uniportal VATS has become a new area of exploration in minimally invasive thoracic surgery resulting in recent improvements in VATS techniques and surgical instruments since 2010 (6). The potential advantages of uniportal VATS include reduced access trauma, less postoperative pain, faster rehabilitation, greater patient satisfaction, and a less invasive approach than conventional VATS techniques (7, 8).

For mediastinal tumors, most surgeons perform thoracoscopic resection via the intercostal or subxiphoid route. However, for brachial plexus schwannomas at the cervicothoracic junction, most surgeons perform open surgery to remove the tumor (9). Zhu et al. (10) compared the perioperative efficacy of transcervical resection with thoracoscopic resection of anterior superior mediastinal tumors. They found that an anterior superior mediastinal tumor treated through a cervical incision is a safer procedure with better perioperative outcomes than VATS. For tumors located at the tip of the thorax, adjacent to the vertebral body, subclavian artery, and/or brachial plexus, some authors prefer nuclear removal rather than complete resection of the tumor due to the risk of recurrence and preservation of brachial plexus function. However, nucleation or intracapsular excision during capsule dissection damages normal fascicles. The combined cervical and uniportal VATS approach is a well-thought-out strategy for this complex surgery. By using the cervical approach to dissect the upper pole of the tumor, the surgeon can create enough space for the thoracoscopic approach to remove the tumor completely. This approach not only ensures the integrity of tumor resection but also helps to solve the problem of limited space, which can be a significant challenge during surgery at the cervicothoracic junction. Moreover, this approach helps to avoid accidental intraoperative bleeding, such as left subclavian artery bleeding, and protects important organs from damage. Thus, it is crucial to devise the most appropriate surgical approach and path to ensure the safe and complete removal of the tumor from this site. In this case, a 21-year-old female patient had no definite pathology before surgery. A posterolateral or “L” incision was abandoned in favor of a neck incision combined with a single intercostal thoracoscopic approach to remove the tumor, with consideration for the cosmetic effect of the incision. This combined approach reduced surgical trauma resulting in a faster postoperative recovery.

Brachial plexus injury can lead to severe sensory and motor dysfunction, affecting a patient’s quality of life. However, Femia et al. (11) recently reported radical resection of mediastinal schwannomas originating in the brachial plexus without neurological complications using intraoperative neuromonitoring. In our case, the origin of the schwannoma from the brachial plexus bundle was determined during surgery and brachial plexus injury was observed without intraoperative nerve monitoring. The postoperative skin temperature on the left side of the patient was lower than that on the right side, and the sensation of the left upper limb was diminished but recovered within one month. Therefore, we advocate for the implementation of intraoperative nerve monitoring to safeguard nerve function and minimize potential damage during the stripping process, particularly in cases involving the use of energy instruments. In addition, three-dimensional reconstruction of tumors is essential for the treatment of mediastinal brachial plexus schwannomas.

In summary, we describe a patient with a brachial plexus schwannoma who was safely excised by combining a cervical incision and an intercostal uniportal VATS approach and demonstrate its feasibility. It is worth noting that this method is a relatively complex procedure that requires special surgical instruments and a good surgical team; safety is the most important consideration.

## Data availability statement

The original contributions presented in the study are included in the article/supplementary material. Further inquiries can be directed to the corresponding author.

## Ethics statement

Written informed consent was obtained from the individual(s) for the publication of any potentially identifiable images or data included in this article.

## Author contributions

LW drafted the manuscript. Data acquisition was performed by LW. LW and LG designed the analysis. YR and LW conceived of and designed the study. All authors contributed to the article and approved the submitted version.
